# Lung Ultrasound and Bioelectrical Impedance Analysis for Fluid Status Assessing Patients Undergoing Maintenance Hemodialysis

**DOI:** 10.1155/2024/1232211

**Published:** 2024-01-09

**Authors:** Danna Zheng, Yueming Liu, Yuting Li, Juan Jin, Qiang He, Xiaogang Shen

**Affiliations:** ^1^Suzhou Medical College of Soochow University, Suzhou, Jiangsu, China; ^2^Urology & Nephrology Center, Department of Nephrology, Zhejiang Provincial People's Hospital (Affiliated People's Hospital), Hangzhou Medical College, Hangzhou, Zhejiang, China

## Abstract

**Background:**

Volume overload is a fatal complication for people undergoing hemodialysis. Therefore, regulating a patient's “dry weight” based on their fluid status is imperative. Clinical experiences are too subjective to accurately judge a patient's fluid status, but techniques have emerged for improved fluid control in the two decades. Specifically, lung ultrasonography (LUS) uses a unique aspect of ultrasound images, the B-lines, to evaluate extravascular lung water, which has increasingly attracted attention. However, the role of B-line quantification in predicting short-mid-term death and/or cardiovascular complications is unclear.

**Methods:**

Patients undergoing MHD at the hemodialysis center of Zhejiang Provincial People's Hospital from October 1, 2020, to February 28, 2021, were examined using LUS and a bioelectrical impedance analysis before and after dialysis, and related clinical data were collected. All patients were followed up for one year after the examination, and deaths and first cardiovascular events (e.g., stroke, myocardial infarction, and heart failure) during this period were recorded.

**Results:**

98 patients were enrolled and divided into three groups in relation to their mild (<16 B-lines), moderate (16–30 B-lines), or severe (>30 B-lines) hypervolemia, defined by the number of B-lines. The long-term survival rate was significantly lower in the severe group than in the mild and moderate groups. LUS and bioelectrical impedance-related parameters (e.g., extracellular water-to-water ratio) were closely related to cardiac ultrasound parameters (left ventricular ejection fraction) (*P* < 0.001). The optimal B-line cutoff value on LUS for predicting fluid overload (defined clinically) in patients on hemodialysis was 11.5 lines (AUC = 0.840, 95% confidence interval 0.735–0.945, *P* < 0.001), and the diagnostic sensitivity and specificity were both 76.5%. During the one-year follow-up period, ten deaths and six cardiovascular events occurred. The survival rate was significantly lower in the severe group than in the mild group (log-rank test *χ*^2^ = 10.050, *P*=0.002) but did not differ between the severe and moderate groups (*χ*^2^ = 2.629, *P*=0.105).

**Conclusion:**

LUS is a cheap, noninvasive, simple, and repeatable volume-monitoring method that can assist with individualized fluid volume management in patients undergoing MHD. LUS results may also help to predict the short-mid-term survival rate of patients to a certain extent.

## 1. Introduction

Most patients undergoing maintenance hemodialysis (MHD) have different degrees of fluid retention, and overhydration increases cardiovascular and cerebrovascular disease prevalence and mortality rates [1, 2]. Therefore, formulating the optimal weight for patients on hemodialysis, called the “dry weight,” is crucial. Currently, clinical parameters, including blood pressure, heart rate, and clinical symptoms, are used to determine the volume status of these patients. However, these parameters are too subjective and changeable to assess the actual hydration condition [3, 4]. Therefore, objective, reproducible, stable, and accurate methods for evaluating a patient's fluid status are urgently needed [5, 6].

Some fluid management methods have emerged, ranging from laboratory examinations to novel technologies [7], for example, atrial natriuretic and B-type natriuretic peptide analyses, lung ultrasound (LUS) exams, and bioelectrical impedance techniques [8, 9]. Specifically, the bioelectrical impedance analysis (BIA) estimates various body composition parameters, such as total body water (TBW) and extracellular water (ECW) volume, to determine a patient's hydration status [10, 11]. Furthermore, BIA is an operational bedside instrument that measures the body's fluid composition by calculating the resistance and reactance reflected by different electric currents [12]; thus, fluid status assessments are more accessible and convenient [6, 13].

LUS is another popular technique for fluid volume assessments in patients undergoing hemodialysis [14, 15], which involves calculating the number of B-lines to estimate the volume of extravascular lung water (ELW). B-lines are comet-tail artifacts created by the pleural line extending to the screen's edge and moving synchronously with breathing behavior [16]. LUS has been used in critically ill patients [17], including those with heart failure [18], respiratory failure [19], and hemodialysis. Moreover, data from the lung water by ultra-sound-guided treatment to prevent death and cardiovascular complications in high-risk end-stage renal disease patients with cardiomyopathy trial (i.e., LUST) suggest using LUS to guide interventions aimed at alleviating lung congestion in high-risk patients on hemodialysis [3].

Currently, radioimmunoassay is the gold standard for evaluating a patient's fluid volume status, which is relatively complex and time-consuming, although it has good accuracy [20]. Some trials have supported using BIA for patients on hemodialysis [20, 21], including observational studies in which overhydration indices assessed by BIA were independent predictors of mortality in hemodialysis [22]. However, the correlation between fluid status assessed by BIA and B-lines detected by LUS remains unclear; some studies have shown a strong correlation between BIA and LUS [11], while others have not [23].

Therefore, this study explored the relationships between LUS and other techniques, such as clinical evaluations and BIA, for assessing fluid status. Additionally, we assessed the discriminatory power of BIA for identifying fluid overload in patients undergoing MHD.

## 2. Methods

### 2.1. Study Population

We conducted a cross-sectional study of all consecutive patients undergoing hemodialysis at the Department of Nephrology of Zhejiang Provincial People's Hospital between October 1, 2020, and February 28, 2021. This study was approved through the local ethics committee of Zhejiang Provincial People's Hospital. The inclusion criteria were hemodialysis vintage of ≥3 months, age >18 years, and treatment with standard bicarbonate dialysis three times weekly. The exclusion criteria for LUS were interstitial lung disease, pulmonary fibrosis, heart failure (New York Heart Association class III-IV), acute pulmonary edema, and vascular, cardiac, and acute infectious complications within the past three months that could mislead the B-line count. Patients with cardiac pacemakers or limb amputations were also excluded from the limitations of BIA.

### 2.2. Clinical Assessment of Fluid Status in Hemodialysis Patients

The ideal dry weight was estimated by experienced hemodialysis nephrologists based on the patient's hemodialysis history and clinical parameters (e.g., symptoms, weight, blood pressure, heart rate, edema, and vascular congestion). Excess weight was defined as the weight gain from the dry weight. Residual weight was defined as the difference between the weight obtained after dialysis and the ideal dry weight.

### 2.3. LUS

LUS was performed using a portable ultrasound scanner (Fujifilm Sonosite Inc., Bothell, WA, USA) with a 2–5-MHz convex probe at the beginning and end of hemodialysis. A 28-position B-line score was adopted to calculate the cumulative number of B-lines as an expression of interstitial pulmonary congestion. Anterior and lateral chest scans were performed on both sides of the chest from the second to fourth (left side) or fifth (right side) intercostal spaces at the parasternal to mid-axillary lines. Two different physicians performed two examinations to assess intra- and interoperator concordance. Each operator was blinded to the clinical and bioelectrical impedance analysis data and ultrasonographic measurements performed by the other operator. A third experienced physician with high expertise in LUS evaluations recorded the data.

### 2.4. BIA

The hydration state and body composition were assessed using a portable whole-body bioelectrical impedance analysis device (InBody S10; InBody, Korea) at the beginning and end of hemodialysis. Four electrodes were placed on both sides of the hand and foot with the patient in the supine position. TBW and ECW volumes were derived from electrical measurements combined with body weight, height, age, and sex based on the manufacturers' equations. Per the instruction manual, an ECW to TBW ratio over 0.390 in patients on hemodialysis is the standard indicator of volume overload. The physician performing BIA was well-trained on the device and was unaware of the clinical and LUS data.

### 2.5. Follow-Up

Patients who underwent LUS were followed up for one year. Death and cardiovascular events (e.g., stroke, myocardial infarction, and heart failure) were recorded during the follow-up period.

### 2.6. Statistical Analyses

Continuous variables are expressed as means ± standard deviations. Between-group comparisons were performed for categorical variables using *χ*^2^ test. Differences in continuous variables across groups were calculated using independent *t*-tests when the variables were assumed to be normally distributed. For nonnormally distributed variables, the Mann–Whitney *U* test was performed. Survival analyses were performed using Kaplan–Meier curves, and survival differences were assessed using the log-rank test. Receiver operating characteristic (ROC) curves were used to measure the sensitivity and specificity of LUS, which served as a potential instrument to assess fluid status. Statistical analyses were performed with SPSS Statistics for Windows, Version 22.0 (IBM Corp., Armonk, NY, USA).

## 3. Results

### 3.1. Patient Demographics

**Figure 1:**
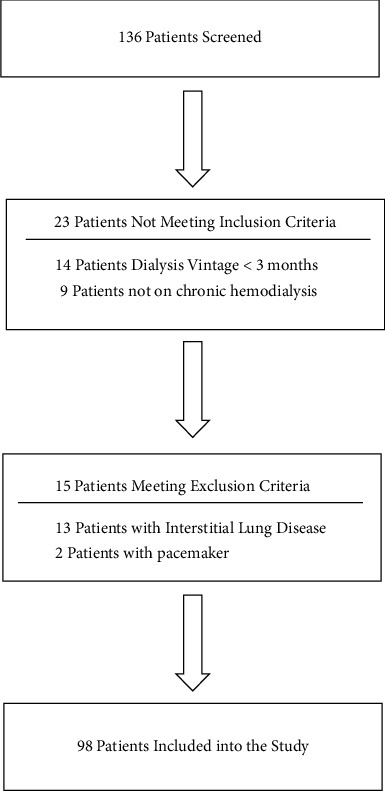
Participant flow diagram for the study.

We enrolled 112 patients on hemodialysis but excluded 14 for limb amputation (*n* = 2), severe lung diseases (*n* = 8), pacemakers (*n* = 2), and acute cardiovascular diseases (*n* = 2) (Figure 1).

Table 1 presents the demographic and clinical characteristics of the study population. Overall, 73 of 98 patients were male (74.49%), and the mean age was 61.46 ± 15.82 years. The enrolled patients were divided into mild (<16 B-lines), moderate (16–30 B-lines), or severe (>30 B-lines) groups based on the number of B-lines obtained from the LUS before hemodialysis; 52, 29, and 17 patients were in the no mild, moderate, and severe groups, respectively. Age, diabetes prevalence, heart rate, systolic blood pressure, albumin level, and ejection fraction significantly differed among the three groups (*P* < 0.05). Systolic blood pressure and heart rate were higher, and the ejection fraction was lower in the severe group than in the other two groups. Dialysis vintage, body mass index, smoking percentage, diastolic blood pressure, hemoglobin level, left ventricular end-diastolic diameter (LVEDD), and left ventricular mass index (LVMI) did not differ among the three groups (*P* > 0.05). The number of B-lines before dialysis positively correlated with LVEDD (*β* = 0.228, *P*=0.030) and negatively correlated with the left ventricular ejection fraction (LVEF; *β* = −0.431, *P* < 0.001) but did not correlate with LVMI (see Supplemental Figure 1).

### 3.2. Ultrasonographic Measurement and Biochemical and Instrumental Parameter Changes before and after Hemodialysis

Table 2 summarizes the ultrasonographic measurement and biochemical and instrumental parameter changes before and after hemodialysis. The number of B-lines, BIA parameters, and body weight significantly decreased after hemodialysis. In contrast, systolic blood pressure, diastolic blood pressure, and heart rate did not differ before and after dialysis.

### 3.3. Correlation between the Change in the B-Line Number and the Ultrafiltration Volume

**Figure 2:**
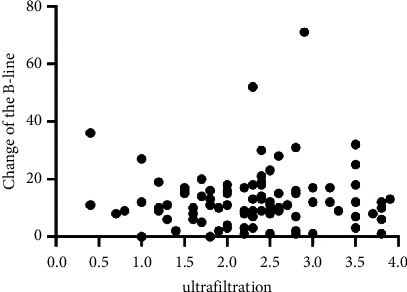
The correlation between the change in the B-line number and the amount of ultrafiltration (liters).

The correlation between the change in the B-line number and the amount of ultrafiltration is controversial. This study did not identify a correlation between the change in the B-line number and ultrafiltration volume before and after dialysis (*r* = −0.043, *P*=0.671) (Figure 2).

### 3.4. Correlations between the Number of B-Lines and BIA-Related Parameters before and after Dialysis

As shown in Table 3, the number of B-lines before and after hemodialysis correlated with some BIA parameters (Spearman's rank correlation analysis). ECW and the ECW/TBW ratio before dialysis (*r* = 0.203, *P*=0.030 and *r* = 0.201, *P*=0.028, respectively) and ECW and the ECW/TBW ratio after dialysis (*r* = 0.223, *P*=0.013 and *r* = 0.189, *P*=0.031, respectively) positively correlated with the number of B-lines. TBW did not correlate with the number of B-lines.

### 3.5. Diagnostic Value of Volume Overload Determined by LUS

**Figure 3:**
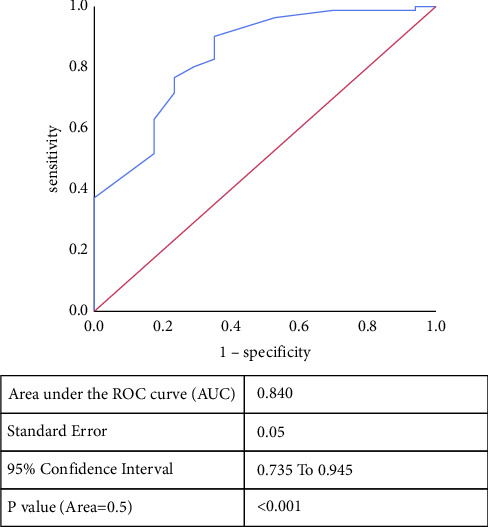
Receiver operative characteristic curve for B-lines identifying volume overload.

To evaluate the predictive power of B-lines using LUS, we used ROC curves for patients on hemodialysis with confirmed fluid retention by BIA (Figure 3). The area under the ROC curve was 0.840 (95% confidence interval, 0.735–0.945; *P* < 0.001). The B-line cutoff value on LUS for predicting overload in patients on hemodialysis was 11.5 lines, which had the best specificity (76.5%) and sensitivity (76.5%).

### 3.6. Survival Analyses

**Figure 4:**
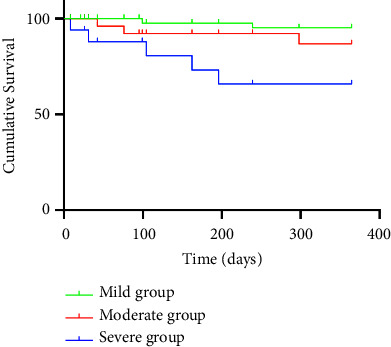
Kaplan–Meier analysis for all-cause mortality according to the number of B-lines.

The enrolled patients were followed for one year after the LUS and BIA exams (Figure 4); 5 of 98 patients were lost to follow-up. During the follow-up period, 6 cardiovascular events (stroke (*n* = 1), myocardial infarction (*n* = 1), and heart failure (*n* = 4)) and 10 deaths (cardiovascular events (*n* = 4), infections (*n* = 3), tumor (*n* = 1), gastrointestinal bleeding (*n* = 1), and overdose (*n* = 1)) occurred; 5 of the patients who died were in the severe group, 3 were in the moderate group, and 2 were in the mild group. The survival rate was significantly lower in the severe group than in the mild group (log-rank test, *χ*^2^ = 10.050, *P*=0.002), but the moderate and severe group rates did not differ (*χ*^2^ = 2.629, *P*=0.105).

## 4. Discussion

This study demonstrates the value of LUS for assessing the volume status of patients undergoing MHD and its relationship with related parameters, such as BIA and echocardiography. Furthermore, we analyzed the relationship between B-lines and long-term patient survival. First, we found that LUS is an important and reliable instrument for assessing the hydration status of patients on hemodialysis. When BIA was used as the “gold standard” for measuring the volume status of patients, LUS showed superior diagnostic performance with high sensitivity and specificity. Moreover, our results indicated that patients with 11.5 or more B-lines could be diagnosed with volume overload, similar to other studies [24]. Second, we found that the number of B-lines closely correlated with BIA- and echocardiography-related parameters but not with ultrafiltration volume and weight changes. Finally, survival analyses with Kaplan–Meier curves demonstrated that the survival rate of the severe group was significantly lower than that of the other two groups, suggesting that B-lines may predict the prognosis of patients with MHD to some extent.

In 2017, nearly 1.2 million people died of chronic kidney disease (CKD) worldwide, making it the 12th leading cause of death [25]. In developed countries, when CKD progresses to end-stage renal disease, the national healthcare system is under enormous pressure because of the medical costs associated with dialysis and kidney transplantation; in developing countries, the increasing number of dialysis patients places a considerable burden on society and the government [26]. Considering the tremendous suffering caused by end-stage renal disease, nephrologists are committed to improving the quality of patients' lives, prolonging their survival time, and continuously improving the quality of dialysis, in which volume control is crucial. Volume overload can result in higher blood pressure and more cardiovascular events, which are often closely related to mortality [27, 28]. Slightly fewer cardiovascular events occurred in this study, and the relationship between the B-line and cardiovascular events could not be further explored. However, reports suggest that volume overload before hemodialysis is a better predictor of patient mortality risk than blood pressure <130 mmHg or >160 mmHg [1, 29]. Nonetheless, volume overload is very insidious, and most patients do not have obvious symptoms, which poses a challenge to clinicians when determining the volume status.

LUS has received the most attention for assessing the volume status of patients on hemodialysis. Regarding diagnoses, the sensitivity and specificity of LUS for volume overload were calculated by ROC curves using the BIA results as the “gold standard.” We found that the specificity was significantly lower than that calculated by Cui et al., with a high false-positive rate [24]. This result may be owing to false “comet-tail signs” due to the thickening of the interlobular septa of the lungs for reasons other than lung disease.

Correlation analyses were an important part of this study. First, we did not identify a correlation between the number of B-lines and the ultrafiltration amount, consistent with some studies [30, 31] but contradictory to the findings of Wu et al. Second, the number of B-lines only represents ELW changes, not systemic volume changes, and is mainly dependent on lung permeability differences. Therefore, the number of B-lines is only weakly correlated with some of the BIA parameters, consistent with the findings of Basso et al. [11, 30]. Finally, some correlations between B-lines and cardiac ultrasound parameters were identified. For example, the number of B-lines before dialysis is correlated with LVEDD and LVEF but not with LVMI. A study from Poland divided patients into LVEF >50% and LVEF <50% groups, finding that the number of patients with >30 B-lines before dialysis was higher in the <50% group than in the >50% group [32]. Although LVEF subgroups were not explored in this study, we found significantly decreased LVEF in the severe group compared to the mild group, severely affecting extravascular volume redistribution. In conclusion, the number of B-lines reflects the volume status and closely correlates with left ventricular function.

To assess the fluid status of patients more precisely, avoiding severe cardiac insufficiency by using LUS is better for volume assessments. This study found that the survival rate of patients in the severe group was significantly lower than that of the other two groups, consistent with other studies. However, this study's follow-up time was short, and its accuracy requires further verification. Furthermore, not only fluid overload is associated with mortality but also intradialytic hypotension episodes and dehydration. Consequently, LUS and B-line quantification should be adopted in clinical practice to reduce long-term mortality also for their ability to prevent intradialytic hypotension episodes and dehydration [33, 34].

Recent studies on lung ultrasound and bioelectrical impedance in assessing the hydration status of hemodialysis patients have overwhelmingly focused on European populations, while fewer studies have been conducted on Chinese populations. Therefore, this study provides a theoretical basis for LUS use in China. However, there are some limitations. First, this was a single-center cross-sectional study with a small number of participants, a short follow-up period, and few endpoint events, which may have caused bias in the results. Therefore, the accuracy of the results requires further examination in a multicenter study with a large sample size. Nonetheless, a randomized controlled study from a single center in Greece showed that the left ventricular size and filling pressures are improved in the group whose dry weight was adjusted based on the number of B-lines [35], which supports our results. Second, patients in this study just accepted LUS examination once; however, one pulmonary congestion cannot represent the average level of B-line number of weeks or months, so it requires more LUS examinations in the future study. Finally, although an ultrasound-experienced clinician performed the LUS exams in this study, and numerous studies have demonstrated high consistency among different LUS operators, operator variability could have influenced the results.

## 5. Conclusions

In conclusion, LUS is a cheap, noninvasive, repeatable instrument to monitor the volume of patients undergoing maintenance hemodialysis, and an ultrasound lung comet sign consisting of 11.5 B-lines, observed upon scanning the upper chest, related to patients with overhydration and lower short-mid-term survival rate to a certain extent.

## Figures and Tables

**Table 1:** Main demographic, clinical, and biochemical data of hemodialysis patients.

Variable	Demographic and clinical characteristics of the enrolled patients			*P* value
	Mild group (*n* = 52)	Moderate group (*n* = 29)	Severe group (*n* = 17)	
Age (yr)	58.96 ± 14.68	69.17 ± 12.93	58.29 ± 18.63	0.009
Male (%)	35 (47.90%)	23 (31.50%)	15 (20.50%)	0.195
Hemodialysis vintage (mo)	26 (10.25, 81)	16 (7.5, 48)	24 (10, 36)	0.336
BMI (kg/m^2^)	22.32 (19.55, 24.41)	22.21 (18.69, 23.55)	20.47 (18.89, 23.21)	0.423
Smokers (%)	11 (47.80%)	6 (26.10%)	6 (26.10%)	0.427
Diabetes (%)	13 (35.10%)	14 (37.80%)	10 (27.00%)	0.017
HR (beats/min)	75.23 ± 11.54	74.35 ± 13.03	83.47 ± 16.35	0.048
SBP (mmHg)	141.19 ± 19.97	155.14 ± 26.06	163.71 ± 18.14	<0.001
DBP (mmHg)	78.77 ± 14.98	78.72 ± 17.03	89.18 ± 17.51	0.056
Hb (g/L)	104.92 ± 14.09	99.28 ± 19.73	103.12 ± 20.2	0.367
ALB (g/L)	38.69 ± 3.77	36.55 ± 3.97	36.61 ± 5.14	0.044
LVEF (%)	65 (62, 67.5)	64 (60, 67.5)	54 (51.5, 58.5)	<0.001
LVEDD (mm)	50.58 ± 6.86	49.59 ± 5.61	53.63 ± 8.96	0.178
LVMI (g/m^2^)	144.23 (115.48, 191.9)	140.96 (113.12, 275.95)	203.25 (124.38, 377.83)	0.245

*Note.* The measurement data are presented as mean ± standard or median (IQR), and the count data are expressed as %. Patients are divided into three groups on the number of B-line. BMI: body mass index; SBP: systolic blood pressure; DBP: diastolic blood pressure; Hb: hemoglobin; ALB: albumin; LVEF: ejection fraction; LVEDD: left ventricular end-diastolic internal diameter; LVMI: left ventricular mass index.

**Table 2:** Changes of ultrasonographic measurements and biochemical and instrumental parameters before and after hemodialysis.

	Pre-HD	Post-HD	*P*
Body weight (kg)	60.91 ± 12.94	58.88 ± 12.75	<0.001
SBP (mmHg)	149.22 ± 23.28	147.19 ± 21.94	0.373
DBP (mmHg)	80.56 ± 16.37	82.09 ± 13.96	0.224
HR	76.40 ± 13.18	77.94 ± 12.38	0.132
B-line	15 (9, 26)	5 (2, 10)	<0.001
TBW (L)	37.29 ± 7.43	35.17 ± 6.84	<0.001
ECW (L)	15.12 ± 3.20	13.77 ± 2.64	<0.001
ECW/TBW	0.40 ± 0.01	0.39 ± 0.02	<0.001

*Note.* SBP: systolic blood pressure; DBP: diastolic blood pressure; TBW: total body water; ECW: extracellular water. The measurement information is expressed as mean ± standard or median (IQR), and the count information is expressed as %.

**Table 3:** Correlation between the number of B-lines and BIA-related parameters before and after dialysis.

	Pre-HD			Post-HD		
	TBW	ECW	ECW/TBW	TBW	ECW	ECW/TBW
Number of B-line	*r* = 0.168 *P*=0.218	*r* = 0.203 *P*=0.03	*r* = 0.201 *P*=0.028	*r* = 0.146 *P*=0.92	*r* = 0.223 *P*=0.013	*r* = 0.189 *P*=0.031

## Data Availability

The data that support the findings of this study are available from the corresponding authors, Shen Xiaogang and He Qiang, upon reasonable request.

## Supplementary Materials

Supplementary Materials Supplemental Figure 1: correlation between the number of B-lines before dialysis and cardiac ultrasound parameters.
