# Supplementary material for: Lung Ultrasound and Bioelectrical Impedance Analysis for Fluid Status Assessing Patients Undergoing Maintenance Hemodialysis
Source: Int J Clin Pract. 2024 Jan 9;2024:1232211. doi: 10.1155/2024/1232211 (PMC10791474; doi:10.1155/2024/1232211)
Supplement: Supplementary Materials — Supplemental Figure 1: correlation between the number of B-lines before dialysis and cardiac ultrasound parameters. [file 1232211.f1.docx]

**Supplemental Figure1.Correlation between the number of B-lines before dialysis and cardiac ultrasound parameters**

**
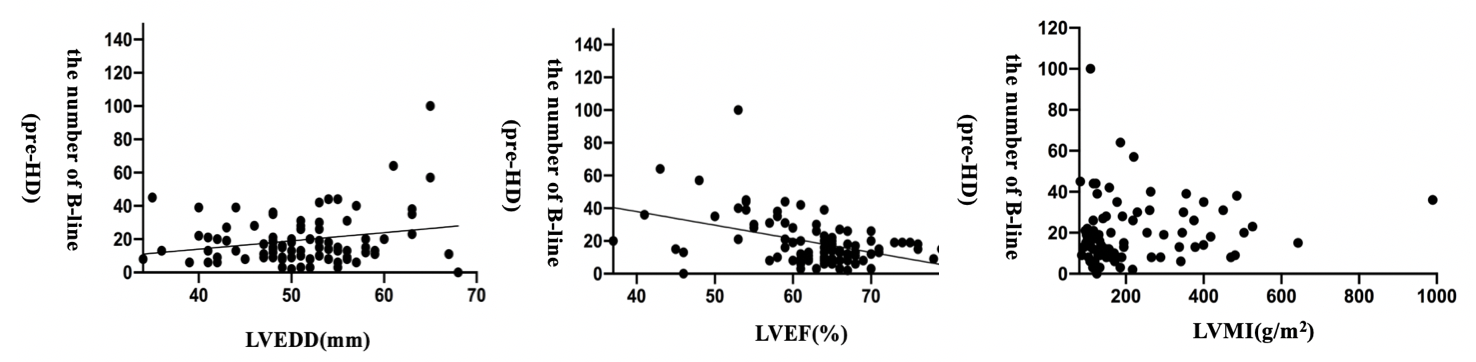
**The number of B-lines before dialysis positively correlated with LVEDD (β = 0.228, *P* = 0.030) and negatively correlated with the left ventricular ejection fraction (LVEF; β = ­–0.431, *P* <0.001) but did not correlate with LVMI.

**The correlation between the number of B-lines(pre-HD) and cardiac ultrasound related parameters.**

Note: EF%: ejection fraction; LVEDD: left ventricular end-diastolic internal diameter; LVMI: left ventricular mass index.
